# Scanning Tunneling Microscope Measurement of Proteasome Conductance

**DOI:** 10.3390/biom15040496

**Published:** 2025-03-28

**Authors:** Sepideh Afsari, Eathen Ryan, Brian Ashcroft, Xu Wang, Stuart Lindsay

**Affiliations:** 1 Biodesign Institute, Arizona State University, Tempe, AZ 85287, USA; safsarim@mainex1.asu.edu (S.A.); eoryan@asu.edu (E.R.);; 2 School of Molecular Sciences, Arizona State University, Tempe, AZ 85287, USA; xuwang@asu.edu; 3 Department of Physics, Arizona State University, Tempe, AZ 85287, USA

**Keywords:** protein electronics, scanning tunneling microscopy, proteasome, electronic sequencing, protein conductance, protein electrical contacts

## Abstract

The proteasome is an enzyme that sequentially degrades peptides into small fragments, so the ability to make electrical measurements of its conformational fluctuations could lead to an electronic readout of the sequence of single peptide molecules. Here, we report scanning tunneling microscope (STM) measurements of the conductance of the *T. acidophilum* 20S proteasome core particle (CP). The wild-type CP did not change conductance significantly as a 4 amino acid peptide substrate was added. Larger peptides were digested by a mutant, CP-Δ12, in which 12 residues were deleted from the N terminus of the alpha chains (opening the central pore). The conductance of this molecule decreased significantly in the presence of a denatured pleiotrophin substrate. Control experiments showed that strong bonding of the protein, both to the substrate electrode and the STM probe, was required for conductivity to be observed. It also appears that substantial penetration of the probe into the protein film is required, a problematic constraint on incorporating the CP into a fixed-gap device for technological applications.

## 1. Introduction

Many redox-active proteins appear to be quite conductive [1,2,3,4,5,6,7,8,9]. This is also the case for proteins that do not contain electroactive cofactors [6], so long as specific chemical connections are made between the electrodes and the protein [10]. A plausible mechanism over distances greater than about 2 nm is hopping between aromatic amino acid residues [11]. Of particular interest are measurements of the conductance of single enzymes [12]. It was shown that the DNA polymerase, Φ29, exhibited significant conductance fluctuations at low bias only when all the components for enzyme activity were present [13]. This raises the question of whether similar fluctuations could be seen in other enzymes that process biopolymers. Here, we focus on the proteasome, an assembly that sequentially digests peptide chains into a range of small fragments [14].

**Figure 1:**
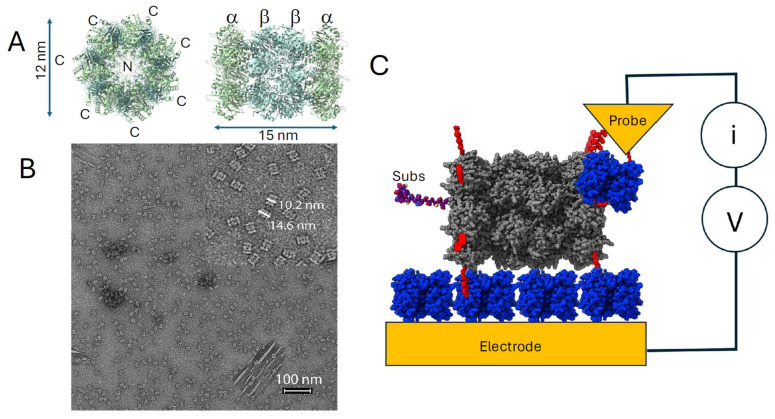
The 20s proteasome and STM measurements. (**A**) Ribbon structure of T. acidophilum 20S proteasome CP (RCSB: 8f6a) looking into the central pore (left) and side-on (right) showing the stack of two α rings and two β rings. The α ring is composed of seven α peptides with their C termini extending out of the ring. The seven N termini point into the central pore. (**B**) TEM image of 20s proteasomes deposited on a carbon film and stained with uranyl formate. Insert is an expanded view showing the dimensions of single molecules. (**C**) The STM conductance measurement. The C termini of the α rings (red) are biotinylated, binding the proteasome (gray) to a streptavidin (blue) coated gold electrode. A streptavidin coated gold probe binds some other biotinylated site on the proteasome to complete the circuit. Measurements are made with, and without, an unfolded protein substrate (Subs).

The 20S proteasome CP [15] from *T. acidophilum* is a cylindrical chamber of four stacked rings (Figure 1A). Two rings at the end are heptamers of the α subunit, while the two rings in the middle are heptamers of the β subunit. The β subunits contain the proteolytic sites while the α subunits act as gates for the proteolytic chamber. When the proteasome is in the inactive state, the α ring is in the “closed” conformation, barring substrate entry. Upon activation, the α subunits undergo a small rotation and the N-terminus of α subunits changes its conformation significantly to allow the entry of substrates [16]. At the entrance point, the α rings may have the most significant interactions with the substrate, making them the logical attachment points for electrodes. In the present work, we express the CP with Avi tag sequences that are subsequently biotinylated using the birA enzyme [17]. As in previous work, [13] both the STM probe and the underlying electrode are coated with streptavidin to facilitate contacts to the biotinylated sites on the CP (Figure 1C). The probe is first pulled back ΔZ nm from a gap, Z_0_, set by a specific tunnel conductance. When a current is detected, the tip bias, V, is swept between ±0.2V and the current is recorded. Many repeats of the process yield a distribution of molecular conductances as described below. Here, we also analyze some of these current–time traces with the goal of better understanding the probe–molecule interaction.

## 2. Materials and Methods

### 2.1. Expression and Purification of Proteasomes

Biotinylation of the proteasome CP was accomplished by appending the Avitag sequence [18] to the C-terminus of the α subunit of the native sequence (WT). The Δ12 variant contained a truncated α subunit with the N-terminal 12 residues (^2^EQGQMAYDRAI^12^) removed. This enlarged the pore of the proteasome and allowed larger peptides to enter the proteasome without the need for an activase [19]. Expression and purification of the Avi-tagged *T. acidophilum* 20S proteasome and its Δ12 variant followed the procedure outlined in Yu et al. [20] Briefly, a petDuet-based expression plasmid containing genes for both the Avi-tagged α subunit and the His-tagged β subunit (Addgene #110805) was transformed into *E. coli* BL21(DE3). The transformed cells were grown in LB at 37 °C until OD_600_ reached ~0.8. The expression of the protein was induced by adding IPTG to a final concentration of 0.5 mM. The induced cells were incubated at 20 °C for ~16 h before harvesting. To purify the proteasome, cells were pelleted at 5000 g for 15 min and resuspended in 30 mL of buffer A (20 mM sodium phosphate, 0.5 M NaCl, 5% glycerol, 10 mM imidazole, pH 8.0) and sonicated. After removing the insoluble material by centrifuging at ~20,000× *g* for 20 min, the supernatant was applied to a 5 mL Cytiva HisTrap column equilibrated with buffer A. The proteasome was eluted with a 5% to 100% buffer B (20 mM sodium phosphate, 0.5 M NaCl, 0.5 M imidazole, pH 8.0) gradient over 15 column volumes. Fractions containing the proteasome were concentrated and purified further using a Cytiva 10 × 300 Superose 6 column equilibrated in 20 mM HEPES, 0.3 M NaCl, pH 7.0 buffer. TEM images (Figure 1B) were obtained on a Talos L120C TEM with samples prepared on carbon-coated copper mesh grids and stained with a 0.7% uranyl formate solution [21].

To biotinylate the proteasome, ~1.5 nmol of proteasome was dissolved in 0.25 mL of buffer containing 5 µg birA biotin ligase, 0.1 M potassium glutamate, 10 mM ATP, 10 mM magnesium acetate, 0.1 mM biotin, and pH 8.0. The mixture was incubated at 30 °C for ~2 h and then kept at ~5 °C overnight. Excess biotin was then removed by exchanging the proteasome into 20 mM HEPES, 0.1 M NaCl, and pH 7.0 buffer. The biotinylated proteasome was concentrated to ~20 uM before being used in experiments.

### 2.2. Preparation of Unfolded Pleiotrophin

Unfolded pleiotrophin (PTN) was used as the substrate for the Δ12 proteasome. To prepare unfolded PTN, recombinant native PTN was produced according to Ryan et al. [22] The native PTN was then denatured using 10 mM DTT to reduce the disulfide bonds essential to the PTN structure. The protein was then exchanged into 20 mM HEPES, 0.1 M NaCl, and pH 7.0 buffer, and treated with 10 molar excess of iodoacetamide to prevent the re-association of the cysteines. Excess iodoacetamide was removed by desalting the protein with 20 mM HEPES, 0.1 M NaCl, and pH 7.0 buffer.

### 2.3. Proteasome Activity Assays

The activity of native 20S proteasome was measured by incubating 16 nM of 20S proteasome with 0.2 mM of suc-LLVY-AMC substrate (Cayman Chemical, Ann Arbor, MI, USA) at 37 °C. Fluorescence of free AMC released by the digestion of the substrate was measured using an excitation wavelength of 380 nm and an emission wavelength of 460 nm (Figure S1). The activity of Δ12 proteasome against unfolded PTN was measured by incubating 100 nM of Δ12 proteasome with 0.2 mg/mL of unfolded PTN in 20 mM HEPES, 0.1 M NaCl, and pH 7.0 buffer. Aliquots of the digest were taken at intervals of 0, 5, 15, 30, and 60 min. The reaction was immediately stopped by adding an equal volume of SDS-PAGE loading buffer to each aliquot. The amount of undigested PTN was measured by Coomassie staining the proteins in each aliquot after SDS-PAGE (Figure S2).

### 2.4. Preparation of Samples for STM

STM tips were fabricated by etching 0.25 mm Au wire (Thermo Scientific, Waltham, MA, USA, 99.999%) using an AC electrochemical method and insulated with high-density polyethylene [23]. For functionalization, the tips were immersed in 50 μM solutions of thiolated biotin (synthesized in our laboratory—see the SI of Zhang et al. [10]) in freshly degassed pure ethanol for three hours. After rinsing with water, the tips were further immersed in a 1 μM streptavidin (New England Biolabs, Ipswich, MA, USA) solution for one hour, rinsed with water, and used immediately.

Gold STM substrates were fabricated by electron-beam evaporation of a 200 nm gold film onto a silicon wafer (Lesker, Jefferson Hills, PA, USA, PVD75) with a 10 nm titanium adhesion layer. Prior to functionalization, the substrates were treated with a hydrogen flame. They were subsequently immersed in 50 μM solution of thiolated biotin in freshly degassed pure ethanol overnight. After rinsing with Milli-Q water (18.2 MΩ), the substrates were incubated in 1 μM streptavidin solution for one hour, followed by a second rinse with water. Finally, the substrates were incubated in a 2 μM protein solution prepared in 1 mM phosphate buffer (PB, pH 7.4) for one hour. For all STM measurements, the 1 mM PB buffer (pH 7.4) used as the covering electrolyte was degassed with argon to eliminate potential interference from dissolved oxygen. The thickness of the monolayer was measured using a Gaertner L123b Ellipsometer (Gaertner Scientific Corporation, Skokie, IL, USA). A refractive index of 1.5 was assumed for the thin organic layer. Measurements were conducted at five different locations on the substrate to calculate the mean monolayer thickness (Table 1). The film thickness increased somewhat with protein concentration but was significantly less than the width of a single molecule (Figure 1A) indicating sparse coverage of the substrate (as also evident in the TEM image, Figure 1B). The unbiotinylated protein also stuck to the substrate, as evidenced by the similar film thickness, but it did not conduct (see below).

### 2.5. Collection of STM Data

STM measurements were performed using a Pico STM (Agilent, Santa Rosa, CA, USA) under electrochemically controlled conditions, with the substrate potential held at 0 V relative to a salt-bridged Ag/AgCl reference electrode [10]. The initial set-point current for all experiments was 4 pA at a bias of 200 mV, corresponding to a distance, Z_0_, above the substrate of approximately 2.5 nm [24].

After stabilization for ~1h in the buffered electrolyte solution, data collection followed the sequence: (1) The Au probe was withdrawn by an additional amount, ΔZ (7 nm unless specified otherwise). (2) The servo mechanism was deactivated, allowing the tip to drift freely. A jump in current to a value > 40 pA was interpreted as the signal for the capture of a protein molecule. (3) Upon capture of the molecule, the voltage bias was automatically swept between ±200 mV at a rate of 0.8 s per sweep, and the corresponding current was recorded as a function of bias (I–V curves). (4) Data were recorded for up to 120 s, after which the tip was withdrawn to the initial set-point current of 4 pA at a bias of 200 mV under servo control. Each data set consisted of over 100 such collections. Very few events > 40pA were observed for values of ΔZ > 7nm. In the absence of the CP, events were only recorded when ΔZ was reduced below 2.5 nm.

## 3. Results

**Figure 2:**
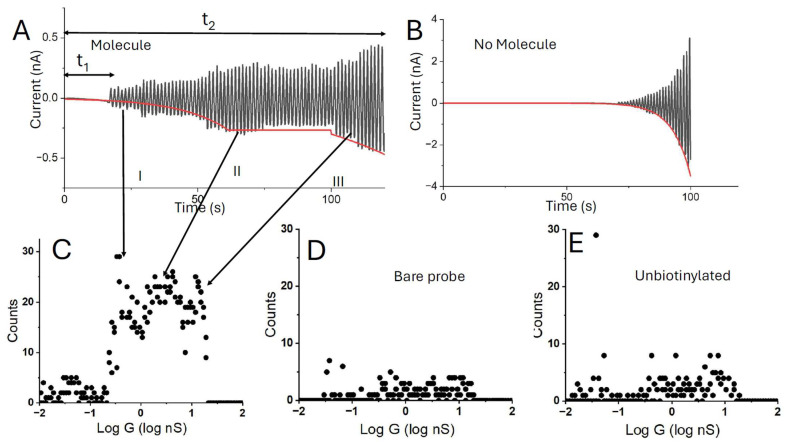
STM measurement process. The probe is first set at a distance of Z_0_ from the electrode corresponding to a current of 4 pA at 0.2V bias (Z_0_~2.5 nm). It is then withdrawn to an additional distance ΔZ (7 nm in this case) and current is recorded vs. time. Once a current >40 pA is detected, the bias is swept ±0.2V producing the sawtooth current shown in (**A**). Initial currents (I) are small owing to non-specific binding, becoming larger and more constant with time as the circuit is completed with specific bonds (II). In some traces, an additional exponential rise is seen (III) as tunneling begins to contribute. The red line is an exponential fit to region I, a constant for region II and a second exponential fit to region III. (**B**) A similar curve for a control sample lacking proteasome. The current–time trace is well-fitted by a single exponential. (**C**) Distribution of measured conductances deduced from the slopes of the current vs. voltage scans for a surface functionalized with the wild-type proteasome. The number of large conductance events is greatly reduced if the tip is unfunctionalized (**D**) or the proteasome is unbiotinylated (**E**).

An example of a current–time trace taken over a sample of WT-CP is given in Figure 2A. It was chosen to show various characteristics of these curves. Common to all these curves is a substantial wait time, t_1_ in Figure 2A, until contact is made. After contact, the current can be quite variable (region I in the Figure) until a region of relatively constant current (region II) occurs. This feature is characteristic of samples that have strong attachment points for the underlying substrate and the probe and is taken to indicate the capture of a molecule in the gap by bonding interactions. This particular trace shows a third region (III) in which the current has an additional exponential growth with time. The corresponding gradients of the I–V curves at each point yield a distribution of conductance values and the results of several hundred measurements are summarized in the distribution shown in Figure 2B. This distribution is broad, with contributions from all three regions of the I–V-t curves.

Consistent with our observations using a number of other proteins [10], bonded connections were required at the two contact points in order for a substantial number of high-conductance traces to be observed. The results of measurements made over a similar time period for a biotinylated WT-CP with an unfunctionalized (bare Au) probe are shown in Figure 2C. Very few high current events are recorded. Similarly, when an unbiotinylated WT-CP is used as the sample, few high current events are observed (Figure 2D) despite the fact that the unbiotinylated WT-CP appears to cover the streptavidin substrate at a density similar to its biotinylated counterpart (as evidenced by the ellipsometry data in Table 1).

**Figure 3:**
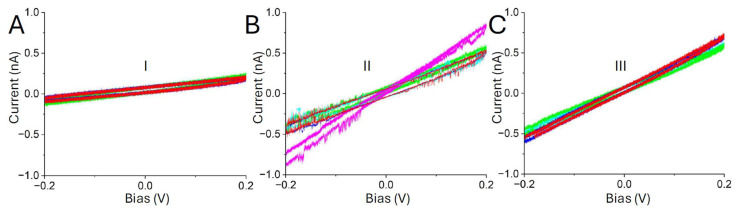
I–V curves corresponding to molecule binding exhibit telegraph noise. (**A**,**C**) shows samples of I–V curves from the region of initial contact (I) and exponential growth (III). Apart from a constant ~20 pA background noise, they are mostly featureless. (**B**) Curves taken in the region of a more constant current (II) display telegraph noise above about 0.1 V, characteristic of these molecular junctions. These curves were selected to show telegraph noise clearly and are not representative of the typical conductances in the three regions (which are shown elsewhere in the conductance histograms).

**Figure 4:**
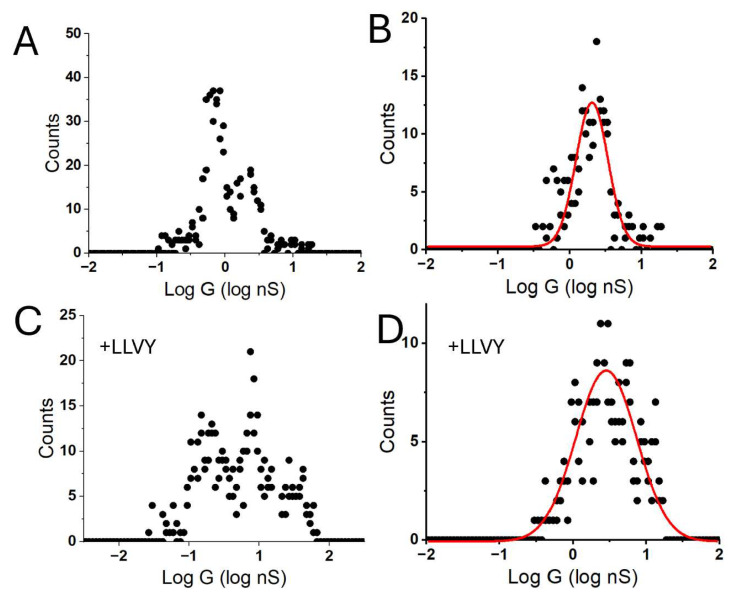
Data filtering. Raw data for the WT proteasome with ΔZ = 7 nm (**A**) and after selection of curves showing telegraph noise (**B**). The filtered distribution is fitted with a single Gaussian (red line). (**C**) Shows raw data for the WT proteasome in the presence of a small fluorescently labeled substrate (suc-LLVY-AMC). (**D**) shows the filtered data, fitted with a single Gaussian, showing that there is little change in the presence of the substrate.

The exponential growth in current in region III of the i-t curve (Figure 2A) is reminiscent of the exponential growth of current with distance characteristic of tunneling, suggesting that in this region, the probe is within 2.5 nm or less of the substrate. Thus, substantial penetration of the protein layer occurs in region III, so the conductances obtained from I–V curves in this region, while corresponding to transport through a connected molecule (connections are required), are also accompanied by distortion of the protein. The curves obtained in region I yield changing conductance values as the tip drifts. It is only in region II that the conductance remains somewhat independent of electrode position as expected for a fixed path through the molecules [25]. This interpretation is further supported by the noise characteristics of the I–V curves. Only in region II do they show the “telegraph noise” (Figure 3) characteristic of these molecular junctions [10,26]. Thus, we have used the presence of this noise to pick out the curves that most likely correspond to a contacted, but less distorted protein. The effects of this selection are shown in Figure 4A,B. This shows a conductance distribution measured for the biotinylated WT-CP in A, with the distribution for the filtered data shown in B. The distribution in B is quite well fitted by a single Gaussian, while the original distribution requires more than one Gaussian for a fit.

Figure 4C,D shows the raw (C) and filtered (D) distributions for a similar sample after the addition of the suc-LLVY-AMC to a final concentration of 5 µM. The filtered distribution appears a little wider after the addition of this substrate, but the peak conductance value is little changed. Table 2 show the peak conductances obtained from single Gaussian fits to the filtered distributions for two different preparations of the biotinylated WT-CP, and for the filtered distribution after the addition of suc-LLVY-AMC. The lack of any substantial change may be a consequence of a transient interaction of the CP with this small peptide substrate.

**Figure 5:**
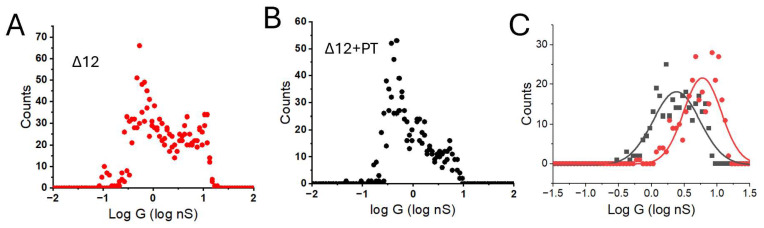
Conductance changes on digesting pleiotrophin. Raw distribution for the Δ12 mutant (**A**), and after the addition of 20 µM denatured pleiotrophin to the same sample (**B**). Filtered distributions are shown in (**C**) for the Δ12 alone (red) and in the presence of the substrate (black). Solid lines are Gaussian fits showing that there is a significant decrease from 6 to 1.7 nS in the presence of the substrate.

Thus, we sought to investigate the processing of longer substrates. We were unable to form complexes of the CP with an unfoldase that remained stable on a surface. For this reason, we changed course to use the Δ12 mutant. The enlarged pore allows for the entry and processing of unfolded proteins [19]. We were unable to demonstrate processing of casein, but found that unfolded pleiotrophin was processed in vitro, as described above. Figure 5 shows the distribution of conductances measured for the Δ12 mutant (Figure 5A) and the same sample after the addition of 20 µM unfolded pleiotrophin (Figure 5B). Filtering of the data as described above produces the distributions fitted by single Gaussians shown in Figure 5C (Δ12 mutant alone, red; Δ12 mutant + pleiotrophin, black). There is a substantial decrease in conductance in the presence of the substrate. These experiments were repeated with three different sample preparations, and, although the conductances varied significantly from preparation to preparation, the drop in conductance after adding the substrate is quite marked (Table 2).

## 4. Discussion

These experiments show that it is possible to measure conductance across some path in the 20s proteasome core particle, and that this conductance changes significantly in the case of the Δ12 mutant in the presence of an unfolded pleiotrophin substrate. In order to consider the implications further, we must first discuss the nature of the STM contact to the molecule.

The approach curves shown in Figure 2 and Figure S3 suggest that the probe must have drifted some 7 nm towards the surface over the total time of the data acquisition, t_2_. In the Figure, t_1_/t_2_ is ~0.5, implying that the probe moved about 3.5 nm towards the substrate before an electrical contact was made, if we assume a constant drift rate. This obvious drift into the tunneling regime is seen infrequently when the microscope is well-stabilized, but a significant wait period before a molecular contact is made is a characteristic feature observed in almost all cases. An analysis of several curves shows a drift towards the surface of ~3.5 ± 0.7 nm, using the exponential growth (region III) as a measure of near-contact. If the set-point, Z_0_, is 2.5 nm, this puts the probe separation from the underlying substrate as around 6 nm when contact is made. This is larger than the diameter of a streptavidin molecule, but much less than the diameter of a proteasome molecule on top of a streptavidin layer. Thus, we conclude that the probe penetrates the protein layer significantly. This probe penetration has been observed in conductance studies of other proteins. The workhorse protein for STM is azurin [27,28,29,30,31]. It has also been studied by conducting atomic force microscopy where the force required to make a conducting contact can be measured. For azurin, the force required to establish a contact is on the order of 10 nN [8,32,33], large enough to result in penetration of the protein film by the probe. This is consistent with a theoretical analysis that shows that substantial deformation is required for a tunnel current to be observed [34].

Conduction at distances larger than the tunneling regime (i.e., region II in Figure 2A) is presumed to occur via hopping [11], for which the conductance, G, is proportional to nD where n is the density of injected charge and D is the diffusion constant for that charge [35]. This leads to the following requirements: (1) Rapid diffusion requires that a path exists via aromatic residues that are spaced by 1.2 nm or less in edge-to-edge distance. (2) It also requires that the energy barriers to hopping be on the order of thermal energy, 3/2 kT. So, if free energy differences are negligible, reorganization energies should be not much larger than 6kT, i.e., no more than a small multiple of 0.15V. (3) Efficient charge injection is needed (there is no intrinsic free charge in a molecular solid). This process is difficult to model [11] but the insertion of an electrode close to an acceptor site is clearly beneficial. Furthermore, close contact with an electrode displaces hydration, likely leading to a reduction in reorganization energies [36].

**Figure 6:**
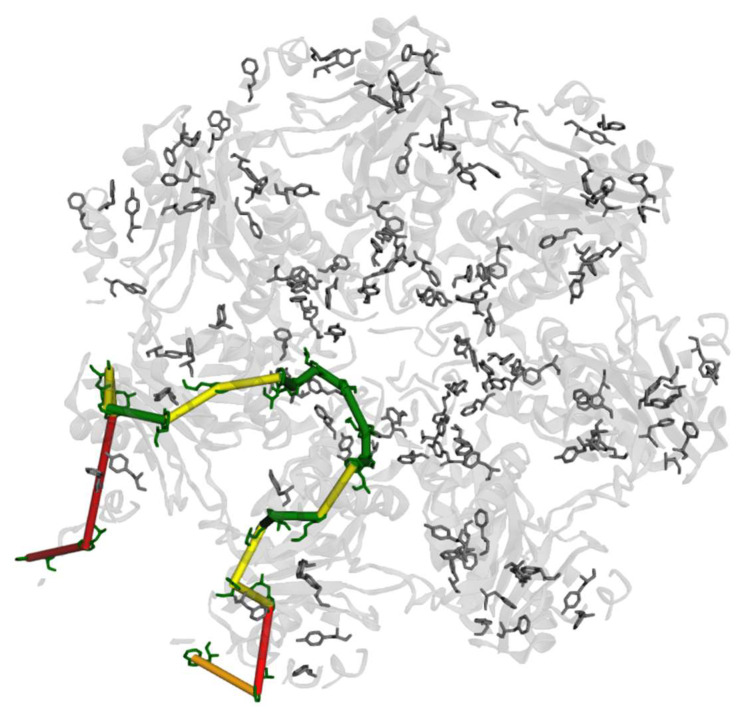
Optimized hopping path from one α peptide and adjacent α peptide. Red is >1.1 nm, orange is 1.1 to >1 nm, yellow is 1.0 to >0.8 nm, and green is <0.8nm edge-to-edge distance.

Here, we discuss only the distance constraints. Examination of the crystal structure of the α ring (Figure S4) shows a significant density of aromatic residues mostly close enough to form a good pathway from one biotinylation site to another on an adjacent α peptide. However, this model is probably not realistic, as it shows the N termini as rigid helices protruding from the structure, whereas in reality, they are known to obstruct the central pore. An AlphaFold3 model with both the N- and C-termini, relaxed by a brief molecular dynamics simulation, is shown in Figure 6. After some molecular dynamics simulations using the program AMBER, both the N-termini and avitags started to unfold. The unfolding of the N-termini closed off the pore as expected, so this is likely a more realistic structure than the unrelaxed one. The path that minimizes hopping distances (calculated using the shortest simple paths function [37]) in this relaxed structure is shown, color coded as described in the Figure caption (distances are listed in Table S1). A similar set of distances is found for a path that traverses the diameter of the protein (Figure S5 and Table S2). The longest (red) jumps occur at the C termini on the outside of the protein (not shown is the distance between the biotinylation site and nearest tryptophan which is about 1.6 nm). This C-terminal region has to be compressed significantly to bring the outer residues close enough for hopping (if free energy and reorganization energies are also favorable).

In the case of the Δ12 mutant, the only aromatic deletion is Y8, and aromatic residues remain densely distributed close to the central pore (Figure S4B), likely accounting for the almost identical conductance of the WT and Δ12. Disruption of this region via insertion of a substrate peptide might account for the large drop in conductance observed (Table 2) in the presence of the denatured pleiotrophin substrate.

## 5. Conclusions

This work shows that significant electronic conductance can be obtained across part of a proteasome molecule, albeit at the expense of what is likely a significant distortion of the molecule. The presence of an unfolded protein substrate leads to a significant reduction in this conductance. It is not possible at this point to say if sequence information might be encoded in this signal. To do this, single molecules would need to be contacted in fixed junctions stable enough to record signals over an extended period. It is not clear how the intrusive contact made by a scanning probe microscope could be replicated in a solid-state chip. This is the principal obstacle to a technology based on interfacing individual protein molecules with solid-state electronics, a field otherwise with great potential [38].

## Figures and Tables

**Table 1:** Film thicknesses measured by ellipsometry for the streptavidin monolayer, two concentrations of the biotinylated core particle (CP) on the streptavidin monolayer and an unbiotinylated core particle on the streptavidin layer.

Sample	Streptavidin	+0.5 µM CP	+1 µM CP	+1 µM Unbiotinylated CP
Thickness (nm)	2.63 ± 0.13	3.85 ± 0.19	4.68 ± 0.62	4.79 ± 0.04

**Table 2:** Conductance at the peak of the filtered distributions for the various samples as shown. Each run is for a different sample preparation except for those labeled * where the same sample was measured before and after the addition of denatured pleiotrophin. Errors are from the Gaussian fit uncertainties. The run-to-run variation is larger.

Sample	G_peak_(Run 1) nS	G_peak_(Run 2) nS	G_peak_(Run 3) nS
WT proteasome	2.69 ± 0.13	2.04 ± 0.1	
WT + LLVY	2.82 ± 0.14	2.57 ± 0.5	
Δ12	4.68 ± 0.23	5.13 ± 0.26	6.02 ± 0.03 *
Δ12 + Pleiotrophin	2.63 ± 0.13	1.99 ± 0.1	1.74 ± 0.09 *

## Data Availability

The raw data supporting the conclusions of this article will be made available by the authors on request.

## Supplementary Materials

The following supporting information can be downloaded at: https://www.mdpi.com/article/10.3390/biom15040496/s1. Figure S1: Fluorescence of free AMC, Figure S2: Coomassie-stained SDS PAGE Gel, Figure S3: A selection of current-time curves, Figure S4: Structure of 3 adjacent α chains, Figure S5: Optimized hopping path, Table S1: Edge to edge distances between aromatic residues in the shortest path connecting two adjacent α chains, Table S2: Edge to edge distances between aromatic residues in the shortest path that traverses the proteasome diameter.
