# Supplementary material for: Scanning Tunneling Microscope Measurement of Proteasome Conductance
Source: Biomolecules. 2025 Mar 28;15(4):496. doi: 10.3390/biom15040496 (PMC12024802; doi:10.3390/biom15040496)
Supplement: Supplementary file 1 [file biomolecules-15-00496-s001.zip › biomolecules-3488843-revised-supplementary.pdf]

Supporting Information for

Scanning Tunneling Microscope Measurement of Proteasome Conductance

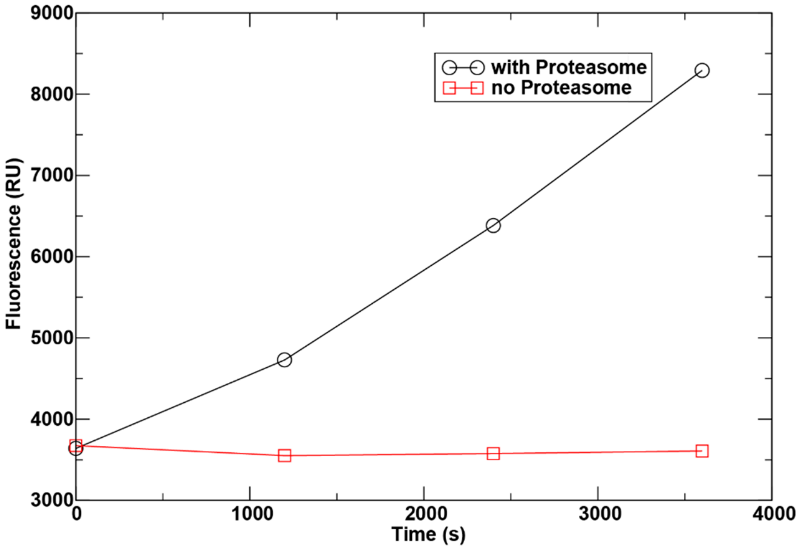

Figure S1: Fluorescence of free AMC released by the digestion of the substrate measured using an excitation wave length of 380 nm and an emission wave length of 460 nm.

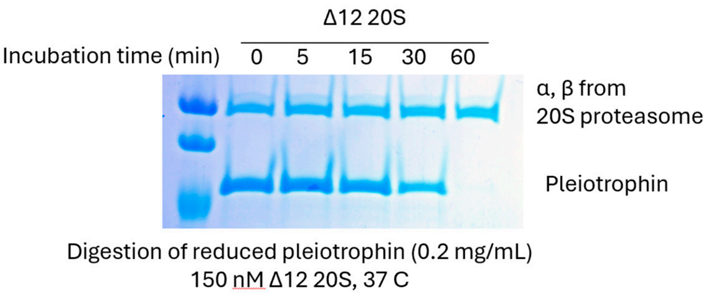

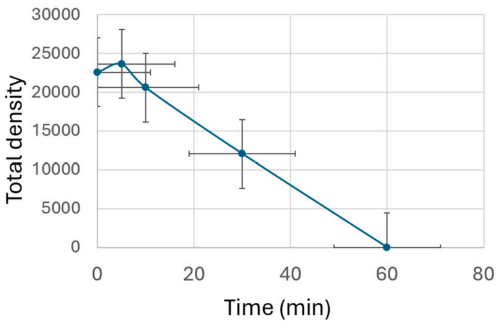

Figure S2: Left: Coomassie-stained SDS PAGE Gel showing the digestion of reduced pleiotrophin (0.2 mg/mL) by the Δ12 20s proteasome (150 nM, 37 C). Right: Optical density

of a gel-band corresponding to intact, unfolded pleiotrophin as it is digested by the Δ12 mutant proteasome.

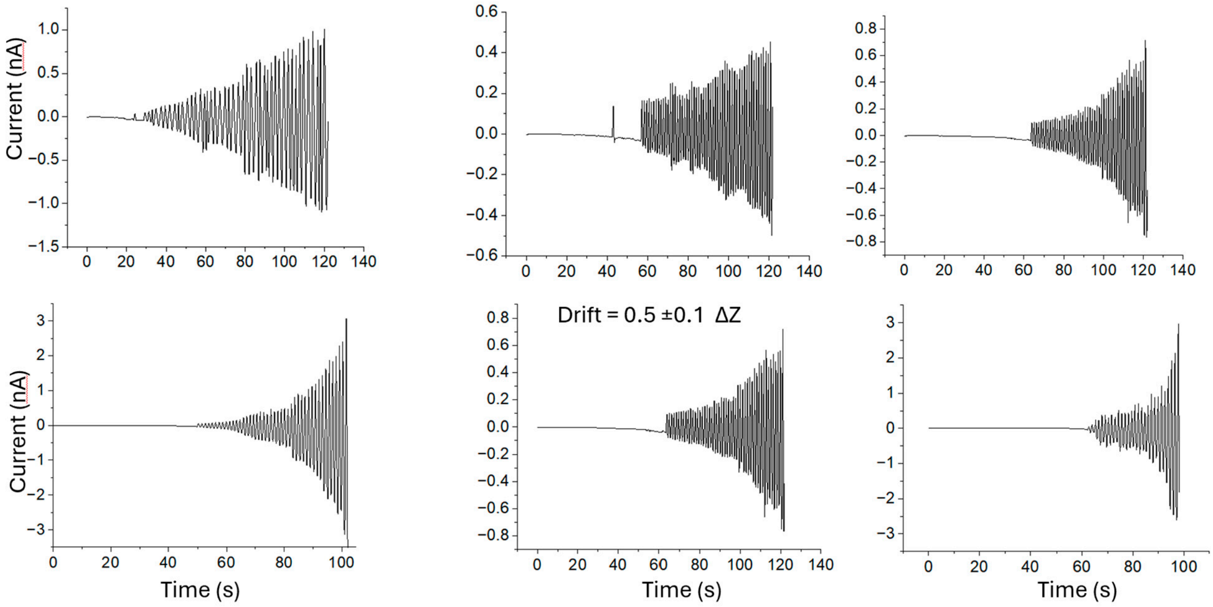

Figure S3: A selection of current-time curves as described in Figure 2 that show the exponential increase in current at the end of the trace. If the time to contact (t_1 in Fig. 2) reflects the amount of drift towards the sample (drift rate =$\Delta Z/t_2$) then the probe has drifted about 0.5$\pm$0.1 ΔZ (N=8) towards the sample to make a conducting contact.

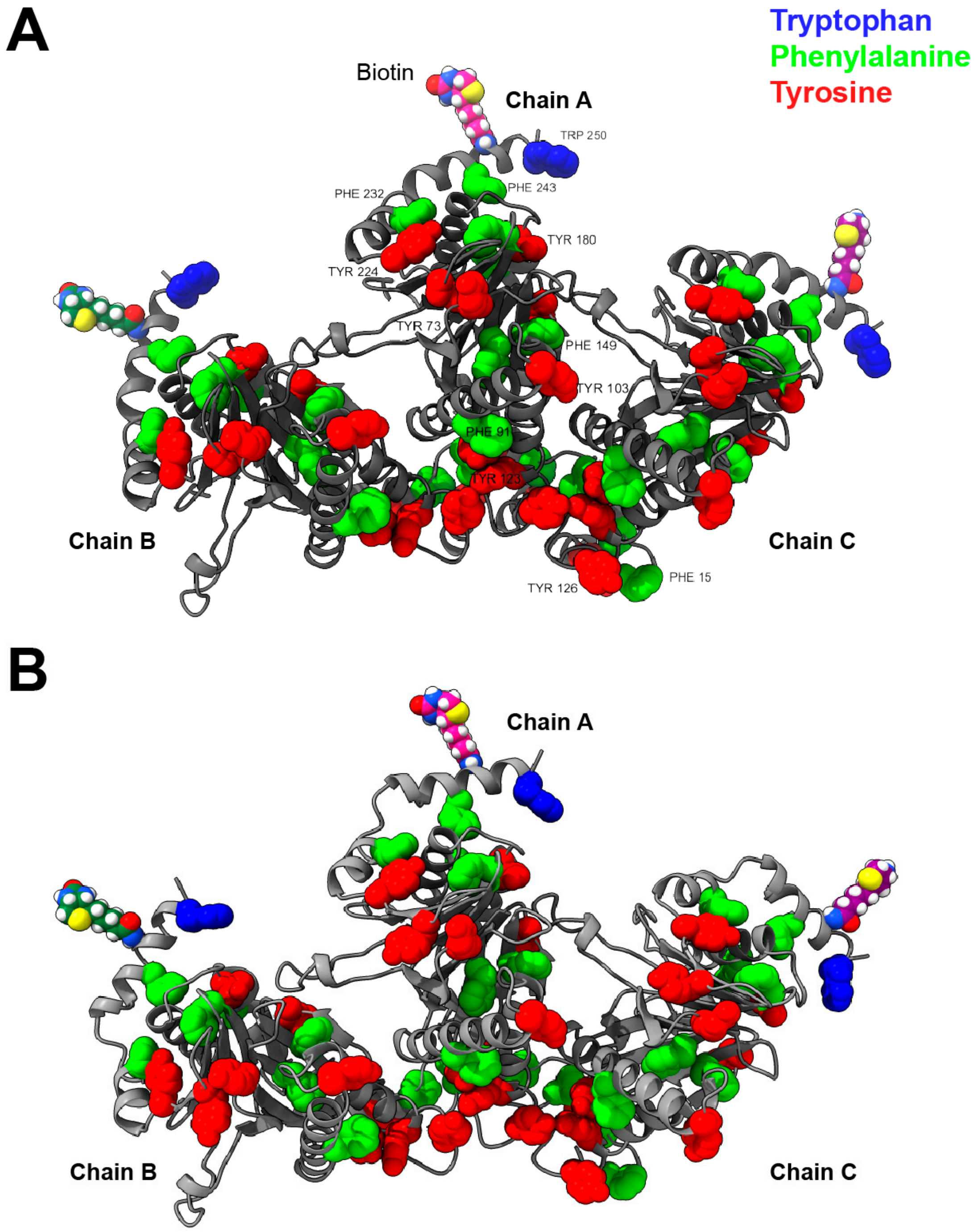

Figure S4: Structure of 3 adjacent α chains with aromatic residues highlighted for A WT-CP and B Δ12 mutant.

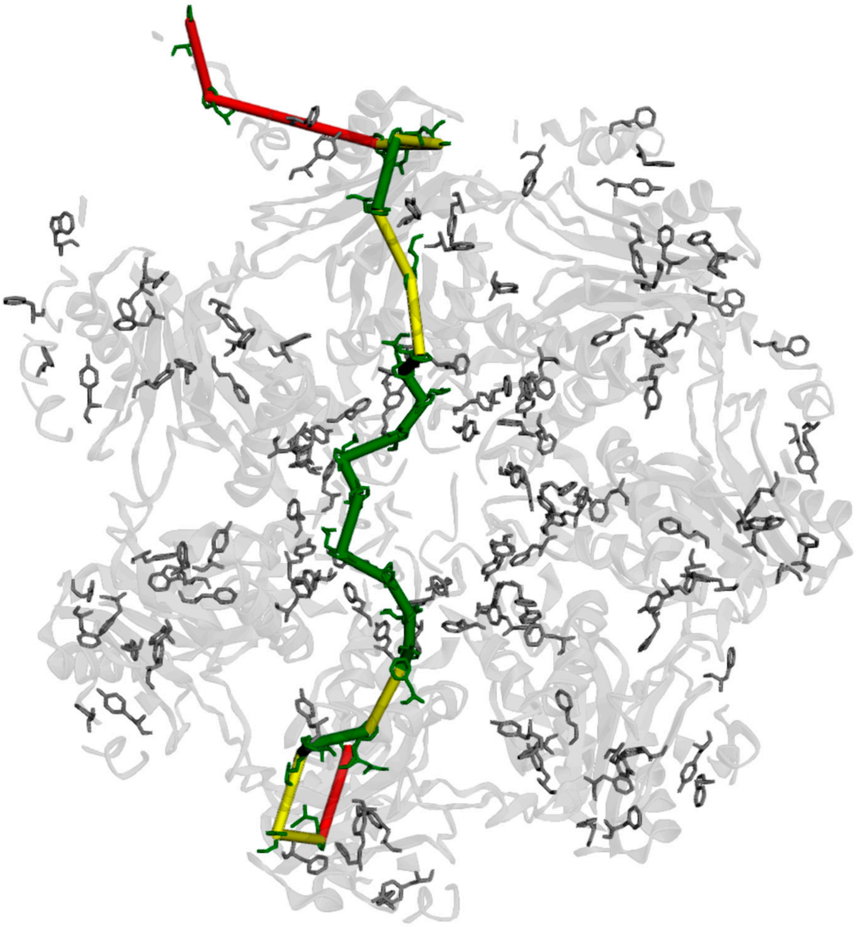

Figure S5: Optimized hopping path that traverses the diameter of the proteasome.

First residue	Second residue	Edge-to-edge distance (nm)
A-W990	A-F983	1.97
A-F983	A-F919	2.56
A-F919	A-Y924	0.93
A-Y924	A-F786	0. 82
A-F786	A-Y965	0.79
A-Y965	A-F882	0.86
A-F882	A-F835	0.82
A-F835	A-Y876	0.75
A-Y876	A-Y867	0.36
A-Y867	A-Y1118	0.37
A-Y1118	A-Y1115	0.63
A-Y1115	A-Y1124	0.33
A-Y1124	A-F1083	0.80
A-F1083	A-F1130	0.83
A-F1130	A-F1141	0.51
A-F1141	A-Y1152	0.67
A-Y1152	A-Y1172	0.95
A-Y1172	A-F1167	0.95
A-F1167	A-F1231	1.25
A-F1231	A-W1238	1.17

Table S1: Edge to edge distances between aromatic residues in the shortest path connecting two adjacent α chains.

First residue	Second residue	Edge-to-edge distance (nm)
A-W990	A-F983	1.97
A-F983	A-F919	2.56
A-F919	A-Y924	0.93
A-Y924	A-F786	0.82
A-F786	A-Y965	0.79
A-Y965	A-F882	0.86
A-F882	A-F835	0.82
A-F835	A-Y876	0.75
A-Y876	A-Y867	0.36
A-Y867	A-Y1118	0.34
A-Y1118	A-Y1115	0.63
A-Y1115	A-Y1366	0.36
A-Y1366	A-Y1363	0.69
A-Y1363	A-Y1614	0.52
A-Y1614	A-Y1611	0.40
A-Y1611	A-Y1620	0.34
A-Y1620	A-F1579	0.73
A-F1579	A-F1626	0.84
A-F1626	A-F1637	0.52
A-F1637	A-Y1648	0.64
A-Y1648	A-Y1668	1.00
A-Y1668	A-F1663	0.89
A-F1663	A-F1727	1.13

Table S2: Edge to edge distances between aromatic residues in the shortest path that traverses the proteasome diameter.
