# Supplementary figures and images for: Scanning Tunneling Microscope Measurement of Proteasome Conductance
Source: Biomolecules. 2025 Mar 28;15(4):496. doi: 10.3390/biom15040496 (PMC12024802; doi:10.3390/biom15040496)

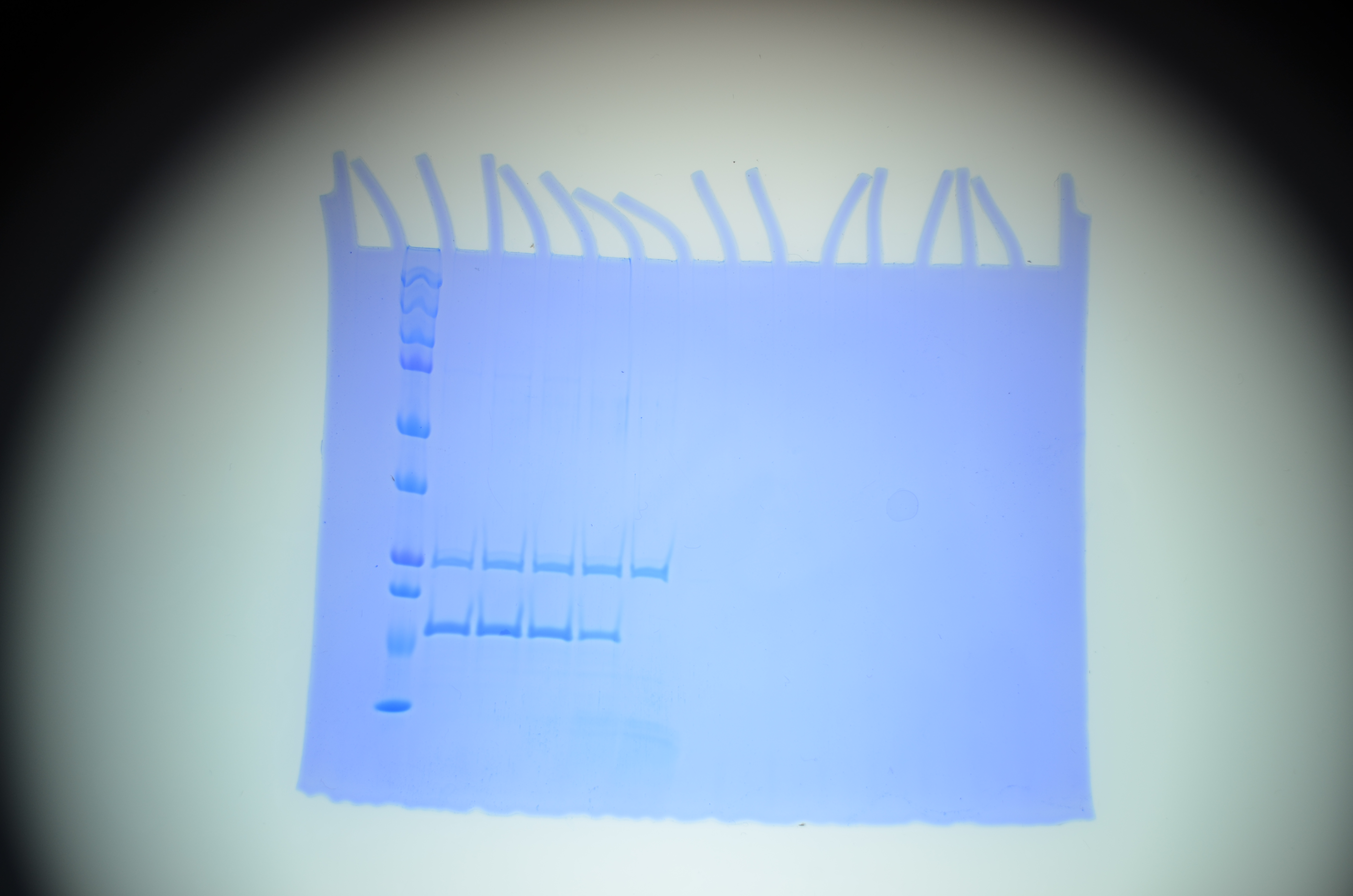

Supplement: Supplementary file 1 [file biomolecules-15-00496-s001.zip › biomolecules-3488843-WB.jpg]
